# Infectious salmon anaemia virus (ISAV) mucosal infection in Atlantic salmon

**DOI:** 10.1186/s13567-015-0265-1

**Published:** 2015-10-21

**Authors:** Maria Aamelfot, Alastair McBeath, Debes H. Christiansen, Iveta Matejusova, Knut Falk

**Affiliations:** Norwegian Veterinary Institute, Oslo, Norway; Marine Scotland Science, Marine Laboratory, Aberdeen, Scotland, UK; Food and Veterinary Authority, Tórshavn, Faroe Islands

## Abstract

All viruses infecting fish must cross the surface mucosal barrier to successfully enter a host. Infectious salmon anaemia virus (ISAV), the causative agent of the economically important infectious salmon anaemia (ISA) in Atlantic salmon, *Salmo salar* L., has been shown to use the gills as its entry point. However, other entry ports have not been investigated despite the expression of virus receptors on the surface of epithelial cells in the skin, the gastrointestinal (GI) tract and the conjunctiva. Here we investigate the ISAV mucosal infection in Atlantic salmon after experimental immersion (bath) challenge and in farmed fish collected from a confirmed outbreak of ISA in Norway. We show for the first time evidence of early replication in several mucosal surfaces in addition to the gills, including the pectoral fin, skin and GI tract suggesting several potential entry points for the virus. Initially, the infection is localized and primarily infecting epithelial cells, however at later stages it becomes systemic, infecting the endothelial cells lining the circulatory system. Viruses of low and high virulence used in the challenge revealed possible variation in virus progression during infection at the mucosal surfaces.

**Electronic supplementary material:**

The online version of this article (doi:10.1186/s13567-015-0265-1) contains supplementary material, which is available to authorized users.

## Introduction

Infectious salmon anaemia (ISA), caused by the ISA virus (ISAV) [1] is an important disease of farmed Atlantic salmon, *Salmo salar* L., and listed as notifiable by the OIE. First reported in Norway in 1984, the prevalence peaked in the 90s, resulting in the implementation of strict biosecurity measures to combat the disease [2]. ISA has also caused significant problems in Scotland, the Faroe Islands, Chile, and on the east coast of Canada and USA. Outbreaks occur as single cases, or localised epidemics. Without any intervention, accumulated fish mortality may reach 80% over several months [3]. No treatment exists and the efficacy of available vaccines is disputed. Anaemia, haemorrhaging in the eyes and skin, pale gills, ascites, dark liver and swollen spleen are common signs in diseased fish suggesting circulatory failure as a major mechanism of pathogenesis (reviewed in [4]).

All aquatic viruses, including ISAV, must cross mucosal barriers [e.g. skin, gills or gastrointestinal (GI) tract] to enter the host. The gills are believed to be the entry port for many infectious agents [5] as they are constantly exposed to the water. Previously we showed that a low virulent ISAV (LVI) infected and replicated more rapidly than a highly virulent virus (HVI) in the gills following immersion challenge, suggesting that the mechanism of entry and early infection phase of ISAV may vary between different isolates [6, 7]. Both endothelial cells and gill epithelial cells may be infected with the virus [8]. In addition, primary gill epithelial cell cultures support ISAV replication [9]. Whilst ISAV infection of endothelial cells is generalized and associated with high mortality [8], infection with low- or putatively non-virulent variants of ISAV (HPR0) has only been detected in epithelial gill cells by IHC (unpublished results) leading to localized and transient infection not associated with the ISA disease [10].

To our knowledge, other entry ports for ISAV such as the skin, eye and GI tract have not been investigated. In mammals or poultry, influenza A virus can use the eye, nose and GI tract as port of entry and primary replication site in addition to the respiratory tract [11, 12], and the virus receptor has been detected in conjunctival cells in humans [13]. The skin of mammals is an effective barrier against viral infections. However, in fish, the outermost epidermal cells remain viable and non-keratinized [14], covered and protected by a mucus layer. In fact, fin and skin have both been implicated as important entry points for other viruses in fish including viral haemorrhagic septicaemia (VHSV), infectious haematopoietic necrosis virus (IHNV) and koi herpes virus (KHV) [15–17]. The presence of the ISAV cellular receptor in epithelial cells of the GI tract, skin and conjunctiva was previously confirmed [8], suggesting they may also be entry routes for ISAV.

The present study investigated the significance of mucosal infection for ISA pathogenesis by revealing alternative entry routes and sites of early viral replication other than the gills. An experimental immersion challenge with two ISAV isolates of different virulence [6] as well as fish collected during a disease outbreak in farmed Atlantic salmon in Norway (2014), were used. Understanding the early primary replication phase at mucosal surfaces and responses may open new possibilities for disease prevention and vaccine development, i.e. vaccines aimed at stimulating the mucosal immune mechanisms.

## Materials and methods

### Organ sampling during immersion challenge and ISA outbreak

Details regarding the virus production, infection challenge and organ sampling procedure are described elsewhere [6]. Briefly, fish were challenged by immersion with either 10^4^ TCID_50_ of highly virulent ISAV Glesvær/2/90 (HVI) or low virulent Can/F679/99 (LVI). Four fish were sampled at 6 h post infection (pi) and on days 1–8, 10, 12, 14, 19 and 23 pi from each challenge group, respectively. Cotton swabs were used to sample skin mucus from the base of the left pectoral fin and placed individually in RLT buffer (Qiagen). Pectoral fin (including skin at the fin base), gill, eye, mid-gut and hind gut were collected in 10% phosphate buffered formalin; pectoral fin (no skin), gill and hind-gut were also stored in 1 mL RNAlater (Invitrogen). Gills were snap-frozen in isopentane (Sigma) chilled in liquid nitrogen. The experimental trial was approved by the Norwegian Animal Research Authority.

In addition, five moribund fish were sampled from a marine site in Norway with a confirmed outbreak of ISA. Autopsy included recording of macroscopic pathological changes. Gill, pectoral fin, pelvic fin, skin from the side-line, hind-gut, mid-gut and conjunctiva (around the eye) were collected in formalin and 1 mL RNAlater. Heart, liver, kidney and spleen were collected in formalin for histological examination.

### Histology, immunohistochemistry and immunofluorescent labelling

The formalin fixed samples were embedded in paraffin and processed using standard histological procedures, stained with haematoxylin and eosin, followed by routine histological examination. All organs samples were examined for the presence of ISAV by immunohistochemistry (IHC) as previously described [18] using rabbit antibodies to recombinant ISAV nucleoprotein (NP) [19] and scored using our previously described IHC ISAV scoring system [6]. Immunofluorescent labelling (IFAT) was performed on acetone fixed cryo-sections as previously described [8, 20] using a monoclonal antibody to ISAV haemagglutinin esterase (HE) [20] and Alexa Fluor^®^ 488 conjugated anti-mouse IgG (Life technologies) for detection. Sections were mounted in SlowFade^®^ Gold (Molecular Probes), and were examined with a Zeiss LSM 710 confocal laser scanning microscope (Carl Zeiss, Germany) using a 63× oil immersion objective and scored [6].

### Real-time PCR

Total RNA was extracted using either the QIAsymphony^®^ RNA robotic system (Qiagen) (challenge material; 5 mg hind gut and gill, 20 mg pectoral fin and skin/mucus swabs) or the RNeasy tissue kit (Qiagen) (ISA outbreak samples; 5 mg) according to manufacturer’s protocols. For hind gut and all ISA outbreak samples, cDNA was synthesized using the TaqMan^®^ Reverse Transcription Reagents kit (Life Technologies, UK) with oligo-d(T)_16_ as described previously [21] and real-time PCR (qPCR) analysis was performed on a Lightcycler LC96 (Roche) as described in [6] using assays targeting ELF and ISAV segment 8 [7]. For skin swabs and pectoral fin, one-step real-time RT-qPCR (Quantitect Probe RT-PCR kit, Qiagen) was performed as outlined in McBeath et al. [7]. Transcription of immune genes type I and II IFN, Mx and γIP were analysed on a Lightcycler LC96 (Roche) for all tissues and statistical analysis was performed as previously described [7]. RNA species specific RT and qPCR to specifically examine replication (cRNA) and transcription (mRNA) was performed according to McBeath et al. [7, 22].

## Results

The number of challenged fish positive for LVI and HVI respectively at each sampling point by different detection methods in all tested organs is shown in Table 1.

**Table 1:** Number of fish positive for ISAV in gills, hind gut, skin/pectoral fin, eye, and skin/mucus.

Day	6 h	1	2	3	4	5	6	7	8	10	12	14	19	23		
Gills	4	4	4	4	4	4	4	4	4	4	4	4	4	4	qPCR	Low
	0/0	0/0	0/4	0/4	2/4	3/1	4/1	4/1	2/2	3/2	4/3	3/na	4/na	1/na	IHC/IFAT	
	4	4	4	4	4	4	4	4	4	4	4	4	4	4	qPCR	High
	0/0	0/0	0/0	0/1	0/3	1/0	3/2	2/2	2/3	4/4	4/4	4/na	4/na	4/na	IHC/IFAT	
Hind gut	0	2	3	3^a^	4	4	4	4	4	4	4	4	4	4	qPCR	Low
	0	0	0	0	1	2	0	3	1	4	3	1	2	0	IHC	
	0	1	0	4	4	4	4	4	4	4	4	4	4	3^b^	qPCR	High
	0	0	0	2	0	0	1	1	1	3	4	3	3	3	IHC	
Skin and/or fin	4	4	4	2^c^	4	4	4	4	4	4	4	4	4	na	qPCR	Low
	0	0	0	0	3	4	4	4	0	4	4	4	4	2	IHC	
	3	3	4	4	4	4	3	4	4	4	4	4	4	na	qPCR	High
	0	0	0	1	3	4	4	4	0	3	4	4	4	4	IHC	
Eye (*n* = 2)	0	0	0	0	0	0	2	0	1	1	2	2	0	0	IHC	Low
	0	0	0	0	0	0	1	1	0	2	2	2	0	0	IHC	High
Mucus	4	4	4	4	4	4	4	4	4	4	4	4	4	na	qPCR	Low
	4	4	4	4	4	4	4	4	4	4	4	4	4	na	qPCR	High

Number of fish positive for ISAV in gills, hind gut, skin/pectoral fin (fin only for RT-qPCR), eye, and skin/mucus swabs with RT-qPCR or IHC/IFAT in fish infected with low and highly virulent ISAV

na not applicable

^a^The “negative” sample was much smaller than the others and produced an ELF Cp 9 cycles higher than expected

^b^Only 3 fish tested

^c^Only 2 fish tested

### Mucosal infection of experimentally challenged fish

#### Skin swabs

**Figure 1:**
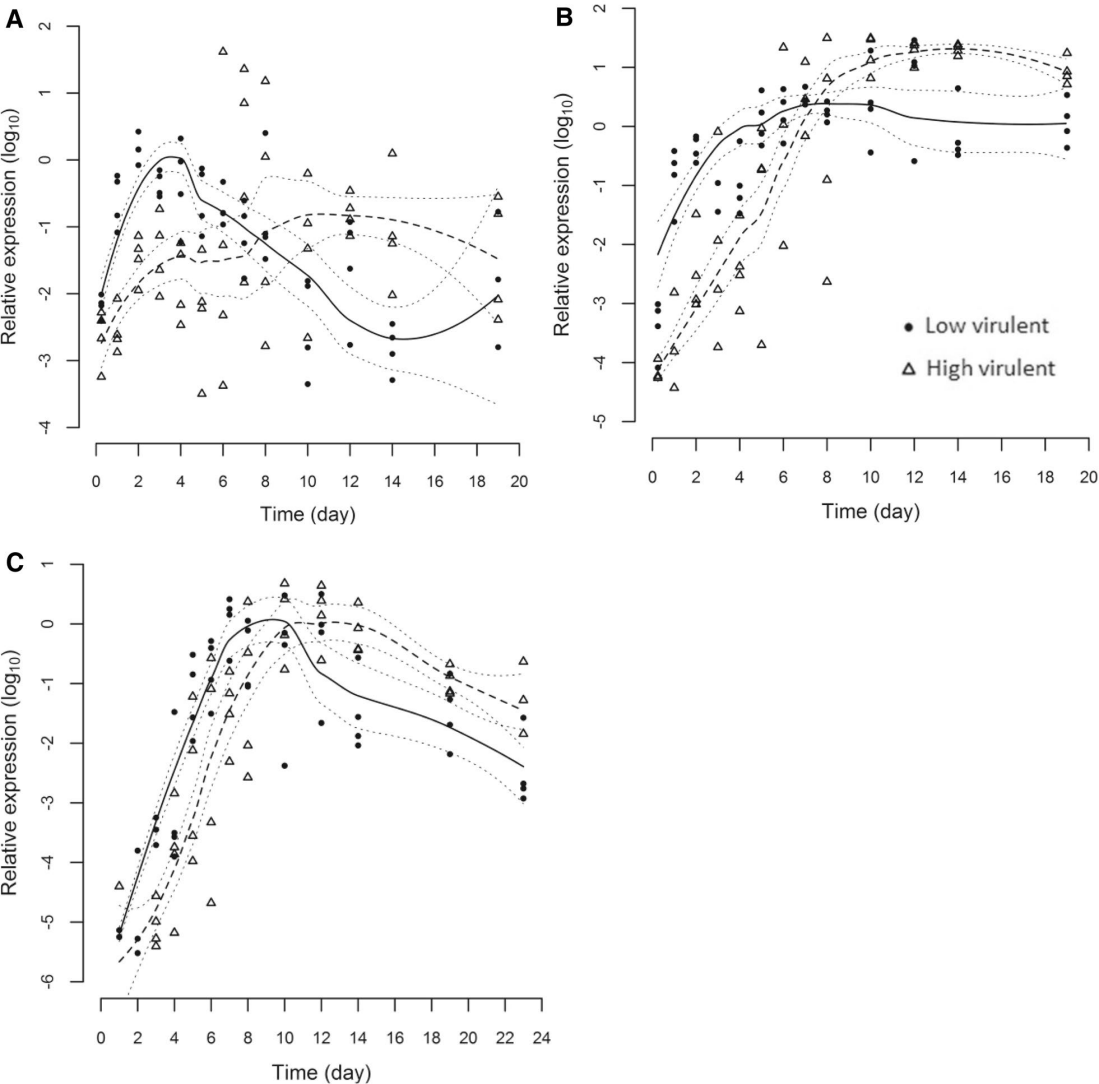
**ISAV segment 8 profiles measured by qPCR**. Statistical modelling of ISAV segment 8 total RNA load profiles measured by real-time RT-qPCR of high virulent (dashed line and triangles) and low virulent (solid line and circles) strains from skin swabs (**A**), pectoral fin (**B**) and hind gut (**C**) post-immersion infection on day 0. Dotted lines indicate 95% confidence intervals.

All skin swabs were positive for virus and replication was demonstrated by a viral load increase in the days immediately following infection (Figure 1A; Table 1). The swabs yielded a mean ELF Ct value of 20.7 (range 18.01–25.16) suggesting the samples contained cells (most likely epithelial cells covering the scales) in addition to mucus. Despite considerable variation in the quantities of virus detected in the individual swabs (as indicated by wide confidence intervals and dispersal of data, including several apparently “super-infected” HVI fish sampled after day 4 pi) a statistically significant difference in dynamics of LVI and HVI was observed (Figure 1A). LVI increased rapidly during the first 4 days pi, with a tenfold increase of virus between 6 h and day 1 pi, indicating early replication, followed by a gradual decrease until the termination of the challenge. Contrary to this, HVI increased slowly in skin swabs throughout the challenge (Figure 1A). The RNA species specific assay detected both mRNA and cRNA in all LVI infected fish on days 2 and 3, but not in HVI infected fish at these timepoints (data not shown).

#### Pectoral fin

Virus was detected by RT-qPCR in all pectoral fin samples except for one HVI fish at each of 6 h and day 1 pi (Table 1). The statistically significant faster increase of LVI (as demonstrated in skin swabs) was also observed in the pectoral fin, further indicating rapid viral entry and replication of this low virulent strain in the epithelial layer (Figure 1B). This was supported by RNA species-specific analysis, although mRNA and cRNA levels only reached detectable levels on day 3 pi for LVI and HVI in 2 and 1 fish respectively. On subsequent days, more LVI- than HVI-infected fish were positive for both RNA types, indicating more rapid replication and transcription (data not shown).

#### Hind gut

Virus was detected in the hind gut by RT-qPCR on day 1 in both HVI and LVI fish (1 and 2 fish respectively, Table 1) but with very high Ct values (>35), indicating low viral load. All fish were positive from day 3 with LVI level increasing faster than HVI up to day 8 (Figure 1C). Both cRNA and mRNA were first detected in hind gut of one LVI fish on day 4 and of 3 fish on day 5 (data not shown). In addition, the measuring of four select immune response related genes indicated little difference in the type I response (IFNα and Mx; Additional file 1). However, the expression of type II IFN and γIP was suggested to be earlier in response to LVI compared to HVI between days 2 and 8 pi (Additional file 1).

### Cell tropism of experimentally infected fish

#### Gills

**Figure 2:**
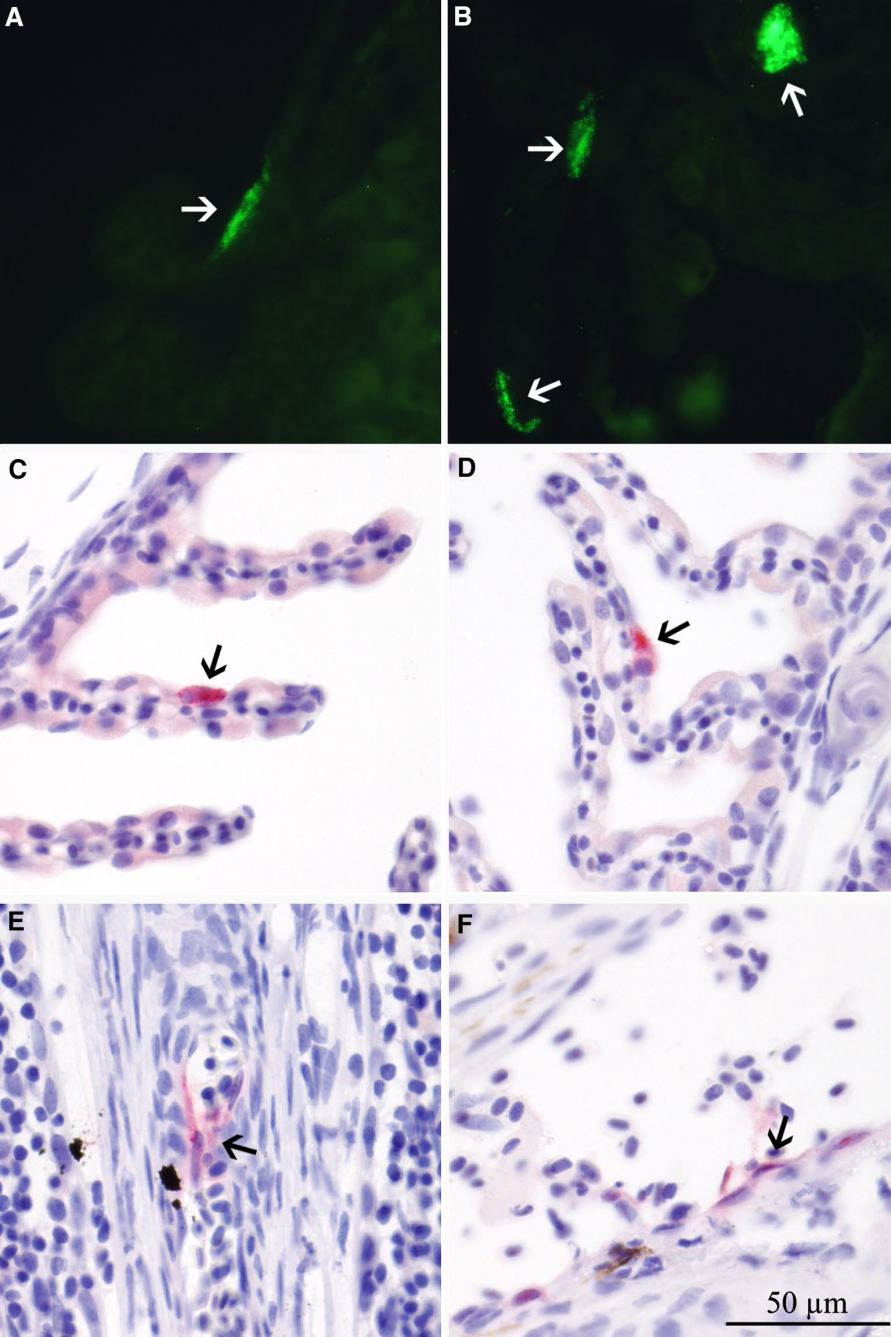
**IFAT and IHC of gills from challenged Atlantic salmon**. IFAT (**A**, **B**) and IHC (**C**–**F**) labelling of gill samples from challenged Atlantic salmon showing cells positive for ISAV HE and NP respectively. **A**, **B** Positive epithelial cells on gill lamellae, day 2 post infection (pi) LVI. **C**, **D** Positive epithelial cells on gill lamellae, day 5 pi LVI. **E**, **F** Positive endothelial cells in the gill, day 6 pi HVI. Arrows show positive cells.

**Figure 3:**
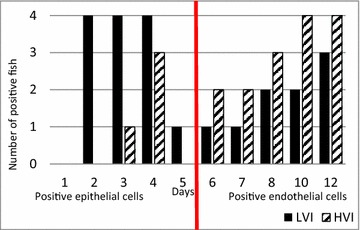
**Number of fish positive for ISAV HE in gill cryo-sections by IFAT**. Fish positive on days 1–5 pi displayed positive epithelial cells, while fish positive on days 6–12 pi displayed positive endothelial cells. A transition from epithelial infection (days 2–5 pi) into endothelial infection (days 6–12 pi) occurs between day 5 and 6.

In general, epithelial ISAV IFAT labelling in cryo-sections (Figure 2) was observed with the amount of positive cells increasing from sparse to moderate between days 2 and 5 pi and scored from 1 to 2 (of maximum 3). Figure 3 shows the number of challenged fish positive for ISAV in the gill cryo-sections and shows the shift from immune staining of epithelial to endothelial cells from day 5 to 6. Epithelial IHC staining was observed between days 4 and 8 pi in the gills from the challenged fish (score 1) (Figure 2). After day 6 by IFAT and day 8 by IHC no epithelial cells were positive for ISAV; however endothelial labelling was observed by both methods.

By IFAT, LVI was detected in epithelial gill cells 1 day earlier (day 2 pi) compared to HVI (day 3 pi) indicating a rapid early establishment of infection for both viruses, but in particular LVI. The number of ISAV positive epithelial cells was low to moderate in the LVI infected fish and scored from 1 to 2 whilst low in the HVI infected fish, all with the score 1. Labelling of endothelial gill cells was observed 1 day earlier in LVI (day 6 pi) compared to HVI (day 7 pi) indicating that LVI established a systemic endothelial infection earlier than HVI, possibly due to the more pronounced primary replication phase in mucosal gill epithelial cells.

#### Skin/pectoral fin

**Figure 4:**
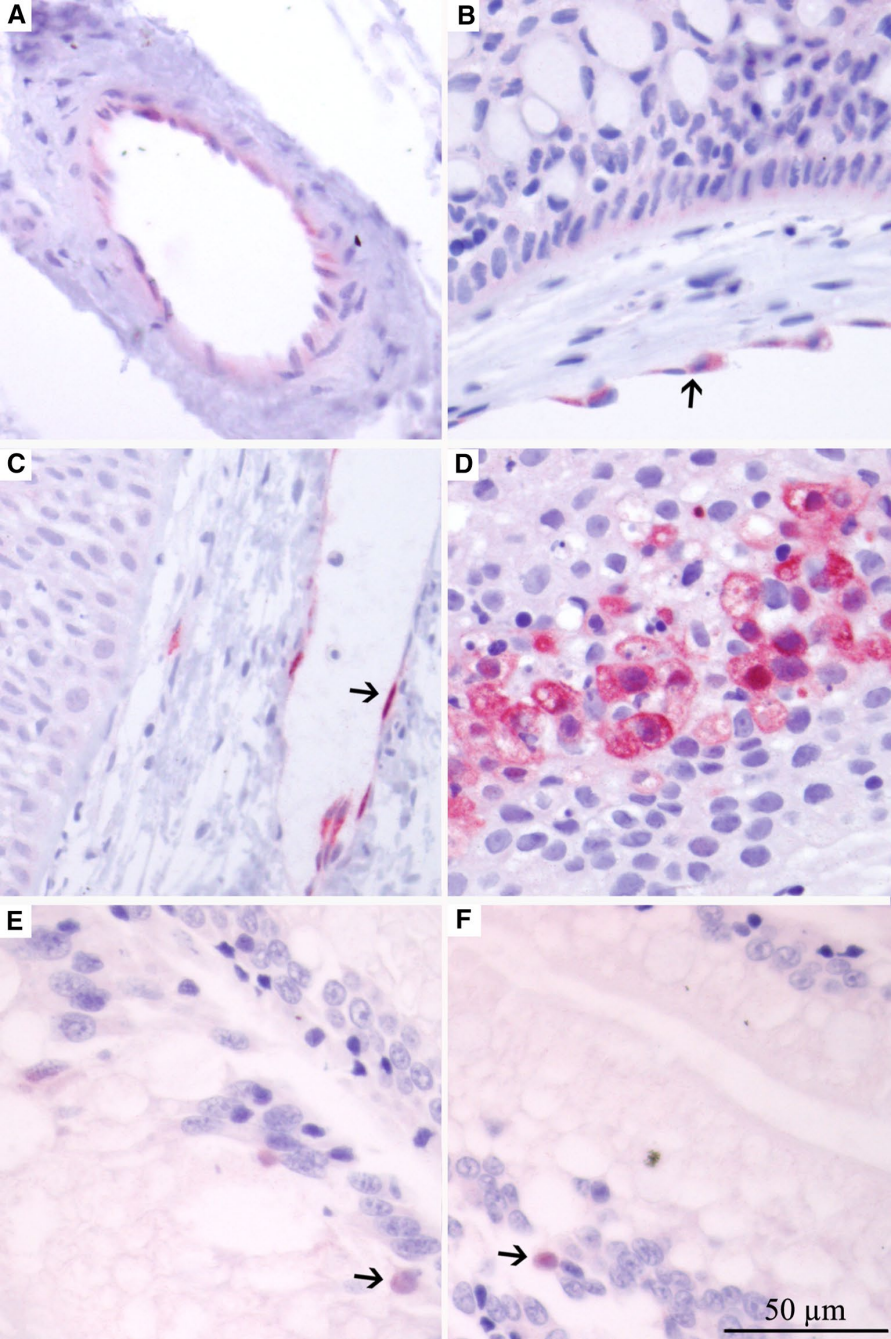
**IHC labelling of pectoral fin/skin and gut from challenged Atlantic salmon**. IHC labelling of pectoral fin/skin and gut samples from challenged Atlantic salmon showing cells positive for ISAV NP. **A** Positive endothelial cells (ECs) in vessel in skin, day 3 pi HVI. **B**, **C** Positive ECs in vessel in skin, day 4 pi HVI. **D** Skin of LVI on day 23 pi, showing necrotic epithelial cells in skin positive for ISAV. **E**, **F** Positive epithelial cells (EPs) in hind gut, day 4 pi LVI. Arrows show positive cells.

ISAV positive epithelial cells were not detected by IHC during the first days pi in the skin/pectoral fin (Table 1). However, large amounts of positive endothelial cells were detected from day 3 pi in 1 HVI fish and in 3 fish from each virus group at day 4 pi. The positive cells [interpreted to be part of the secondary vascular system (SVS)] were found close to the epidermis (Figures 4A–C) and scored from 1.5 to 2.5. From day 4 all fish in both groups had positive endothelial cells in the skin. At day 23 pi positive necrotic epithelial cells (Figure 4D) were found in 1 fish infected with LVI showing that epithelial skin cells can become infected by the virus. Macroscopic skin haemorrhages were observed in the positive fish.

#### Hind gut and eye

In the hind gut, the first ISAV observations by IHC were in epithelial cell nuclei at day 3 pi for the HVI and at day 4 for the LVI. Examples of positive cells are shown in Figures 4E and F. Endothelial labelling was observed from day 6. In the eye, only endothelial cells were positive and only after the virus had been detected in internal organs (Table 1). This suggests the positive endothelial cells in the eye were infected through the cardio vascular system and not as a result of primary viral entry.

### Naturally infected fish

**Figure 5:**
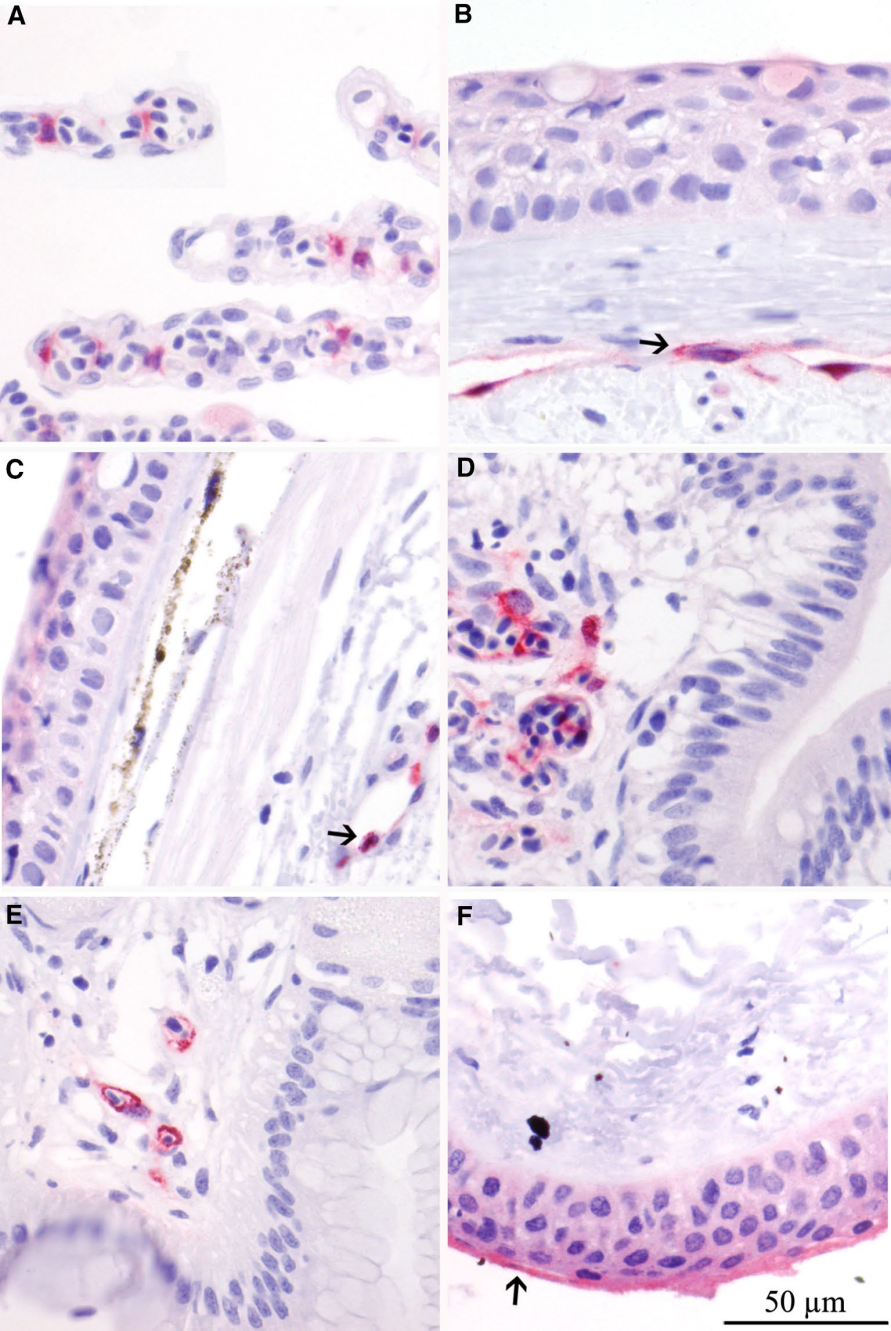
**IHC labelling of mucosal tissues from Atlantic salmon from outbreak**. IHC labelling of mucosal tissues from Atlantic salmon from confirmed outbreak of ISA showing cells positive for ISAV NP. Gill (**A**), pectoral fin (**B**), side-line skin (**C**), hind gut (**D**) and mid gut (**E**) with ISAV positive endothelial cells. Conjunctiva (**F**) with positive epithelial cells. Arrows show positive cells.

**Figure 6:**
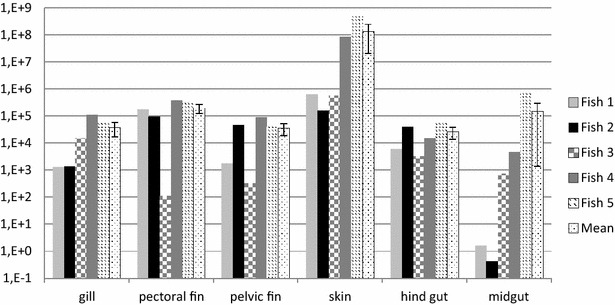
**ISAV segment 8 measured by qPCR**. Log of RT-qPCR ISAV segment 8 load in fish 1–5 from outbreak of ISA standardised to ELF and mean of 5 fish with standard error.

All five Atlantic salmon from the ISA outbreak were anaemic and showed typical clinical and pathological signs of ISA. Histological examination revealed haemorrhaging in several organs and hepatic necrosis associated with ISA. IHC labelling of the mucosal organs revealed positive endothelial cells in gills, pectoral fin, skin, mid-gut and hind-gut (Figure 5). Positive epithelial cells were found in conjunctiva only. All fish were highly positive by IHC (score 2.5–3) and RT-qPCR in all organs tested. Notable was the significantly higher viral load in the skin/side-line samples (Figure 6).

## Discussion

Here we demonstrate for the first time that ISAV was taken up and replicated in mucosal surfaces other than gills, such as the pectoral fin, skin and GI tract. We also present evidence of a shift in cell tropism, from the initial infection of epithelial cells to endothelial cells as the disease progressed, suggesting a primary replication phase in mucosal epithelial cells. Finally, we present observations suggesting a role for the secondary vascular system (SVS) in ISA pathogenesis.

Mucosal surfaces are significant barriers to viral infection. In the present study, following an immersion infection, detection of ISAV in the skin swabs after only 6 h pi indicated an early accumulation of viral particles in the mucus and skin. Interestingly however, the significant temporal increase in viral RNA after 6 h pi indicated ISAV was indeed infecting cells contained in the mucus and actively replicating as confirmed by RNA specific assay. Taken together, this strongly suggests ISAV uses the skin and fin epithelial cells as entry points and sites of primary replication. To our knowledge this is the first description of ISAV using this port of entry in Atlantic salmon.

The infection pattern in the pectoral fin by RT-qPCR was very similar to that seen in gill [6, 7] indicating that the pectoral fins also play a part in the initial infection. Even though the present study used only pectoral fin and skin to examine virus load on outer mucosal surfaces, we speculate that the whole mucosal surface plays a role in early infection. This is supported by live fish real time infection studies with IHNV and VHSV in salmonids and KHV in carp where skin and fin play a role in uptake and primary infection [15–17]. Due to variable sensitivities of the differing assays, virus production in both gill and fin was likely occurring at undetectable levels using IHC and IFAT prior to the positive timepoints, as indicated by the increase in virus level by RT-qPCR. The sample preparation procedures may also have caused loss of surface epithelial cells during histological processing, putatively explaining why no positive epithelial cells were found in the pectoral fin/skin by IHC at early timepoints. Unfortunately no frozen skin or fin were sampled to confirm that the positive RT-qPCRs were indeed caused by epithelial infection and this therefore remains an important aspect for future studies.

Interestingly, the cell tropism and kinetics of the mucosal infection of LVI and HVI appeared to be different. Our previous study indicated a primary replication phase in the gills for the LVI, that was not observed for the HVI [6, 7]. Here we showed that the LVI specifically infects epithelial cells earlier and with a higher score than the HVI, suggesting epithelial tropism. At early timepoints the LVI appears similar to the non-virulent ISAV HPR0, which is largely detected in gill epithelial cells and does not progress into an endothelial infection as demonstrated by IHC examinations (unpublished results), even though it has been detected in kidneys in some cases by qPCR [10, 23]. HVI does not appear to be as active in the epithelial cells, however at the later stages it establishes an extensive endothelial infection leading to higher viral load and higher mortality [6].

The vascular system in fish is divided into two components, the primary vascular system (PVS) containing the red blood cell (RBC) circulation, and a secondary vascular system (SVS) consisting of thin-walled capillaries of varying sizes, not containing RBCs under resting conditions. The SVS is a large-volume low-flow system connected to the PVS through anastomoses, and has only been found in mucosal surfaces in direct contact with surrounding water, e.g. gills, skin, fins, and oral mucosa. The volume of SVS was reported to be 1.5 times the PVS in rainbow trout, *Oncorhynchus mykiis* (Walbaum) [24]. Given that endothelial infection in the skin, interpreted to be part of the SVS, was found preceding IHC endothelial detection in any other organ indicates the cells in the skin were not infected through the cardiovascular system. Thus, we speculate that HVI may have a primary replication phase in the SVS. Furthermore, the significantly higher viral loads in samples from the side-line skin in outbreak fish could also indicate a role of the SVS in ISA pathogenesis. The functional role of the SVS is poorly understood, although ion/water balance, oxygen uptake, acid–base regulation, and communication between the systemic and mucosal protective systems have been suggested [25]. The further exploration of the SVS in ISA pathogenesis is a topic for future studies.

Detection of HVI in endothelial and, only to a limited extent, in epithelial cells at early stages suggests HVI, to a greater extent than LVI, enters directly through the epithelium through transcytosis, thus reaching the endothelial cells underneath. This strategy is one of a variety of mechanisms used by viruses to gain entry to the host [26] and was seen studying VHSV on polarized epithelial rainbow trout cultures [27]. Low virulent strains infected the epithelial layer, while the highly virulent strains passed straight through without replication. LVI on the other hand infects the mucosal epithelium. This may give the fish infected with LVI an advantage by priming the local mucosal immune system, potentially triggering an early systemic protective response, before virus overflows the circulation [4].

Statistically modelled profiles of LVI and HVI in the hind gut were similar to those observed in heart [6]. As previously suggested for the heart, the earlier increase of LVI in the hind gut may be attributed to the more rapid infection of epithelial cells which systemically alerted the immune system. This can be seen in two of the immune markers tested (Additional file 1). The RT-qPCR positives on days 1 and 2 pi may be a result of ingested virus however further work is required to understand the role of the GI tract for ISAV uptake and infection in Atlantic salmon.

Virus shedding was not a focal point of this study however the continuous circulation of new water combined with an increase in the amount of ISAV in the skin swabs from the challenged fish, indicate that the skin may take part in shedding. A high level of virus particularly in the skin of the outbreak fish supports this. Positive epithelial cells in the conjunctiva of the outbreak fish may suggest its involvement in dispersal of virus at a later stage of infection however this should be investigated further. Individuals vary substantially in their responses to infection and this influences the host’s ability to limit or clear the infection. More susceptible hosts are likely to shed more virus than a less susceptible host. A study using IHNV and VHSV in rainbow trout showed that even under substantially controlled conditions, between-individual variation in shedding was prevalent [28]. The considerable variation in the quantities of virus detected in the individual skin swabs, in particular for the HVI, could simply be due to unequal swabbing during sampling however may indicate that not all fish shed comparable levels of virus.

In conclusion, we show for the first time that ISAV uses the skin and pectoral fin as entry ports and primary replication sites in addition to the gill. As the infection progresses, the type of cells infected with the virus shifts from epithelial to endothelial, resulting in a shift from a localized to a generalized infection. LVI and HVI appear to have a different mechanism or pathway of infecting host cells with the LVI having a greater affinity for epithelial cells in the gill, skin and fin while the HVI appears to directly infect endothelial cells leading to more pronounced systemic infection.

## Additional file

10.1186/s13567-015-0265-1 **Statistical modelling of 4 immune marker expression profiles.** Statistical modelling of 4 immune marker expression profiles, type I IFN (A), Mx (B), type II IFN (C) and γIP (D) in hind gut of fish infected with high virulent (dashed line and triangles) or low virulent (solid line and circles) strains measured by real-time RT-qPCR. Dotted lines indicate 95% confidence intervals.

## Abbreviations

ECs: endothelial cells
EPs: epithelial cells
GI: gastrointestinal
HE: haemagglutinin esterase
HPR0: putatively non-virulent variants of ISAV
HVI: highly virulent ISAV
IFAT: immunofluorescent labelling
IHC: immunohistochemistry
IHNV: infectious haematopoietic necrosis virus
ISA: infectious salmon anaemia
ISAV: infectious salmon anaemia virus
KHV: koi herpes virus
LVI: low virulent ISAV
NP: nucleoprotein
pi: post infection
PVS: primary vascular system
SVS: secondary vascular system
VHSV: viral haemorrhagic septicaemia virus
