# Supplementary figures and images for: Infectious salmon anaemia virus (ISAV) mucosal infection in Atlantic salmon
Source: Vet Res. 2015 Oct 21;46:120. doi: 10.1186/s13567-015-0265-1 (PMC4618535; doi:10.1186/s13567-015-0265-1)

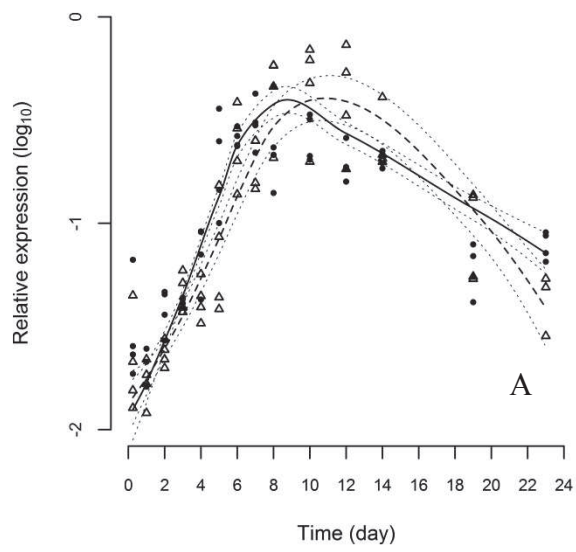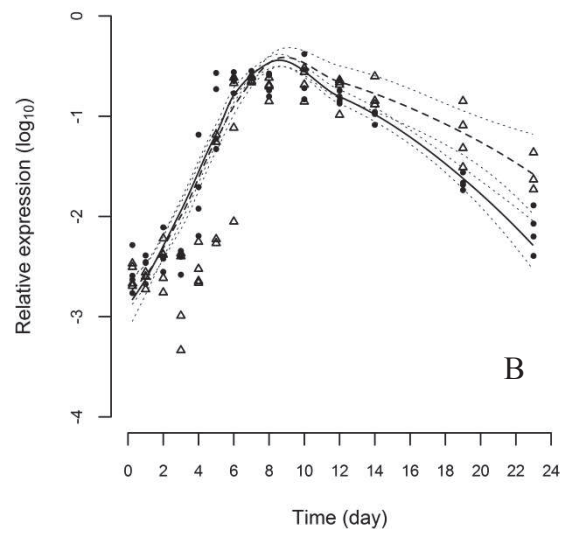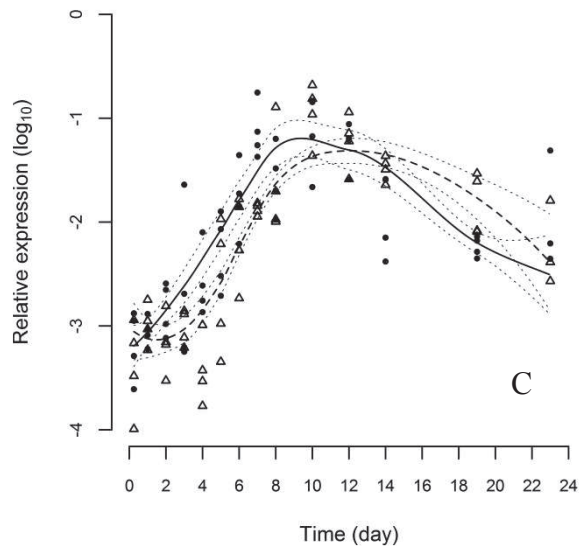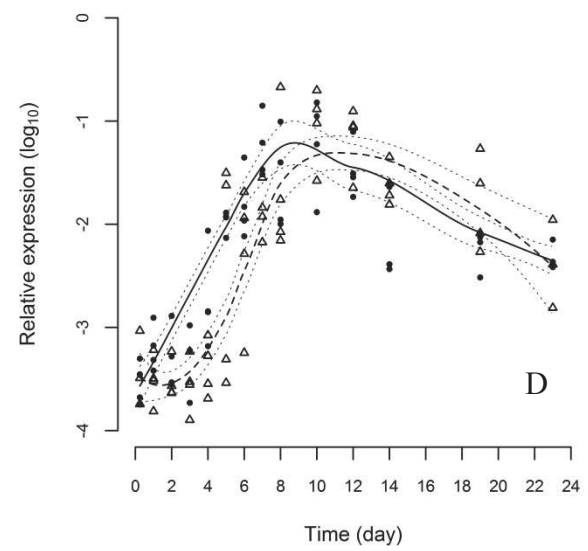

Supplement: Supplementary file 1 — 10.1186/s13567-015-0265-1 Statistical modelling of 4 immune marker expression profiles. Statistical modelling of 4 immune marker expression profiles, type I IFN (A), Mx (B), type II IFN (C) and γIP (D) in hind gut of fish infected with high virulent (dashed line and triangles) or low virulent (solid line and circles) strains measured by real-time RT-qPCR. Dotted lines indicate 95% confidence intervals. [file 13567_2015_265_MOESM1_ESM.pdf]
